# The Groundwork of Eugenics

**Published:** 1909-05-22

**Authors:** 


### THE GROUNDWORK OF EUGENICS.

We certainly think that the appearance of a body of students calling themselves "Eugenists" is as opportune as their name is barbarous. Professor Karl Pearson, who may be looked upon as their governing director, has been at considerable pains to set forth, lucidly and in non-technical language, the objects of the workers at the Eugenics Laboratory. He states, as the foundation of the Eugenic structure, the following claims: (1) "We depart from the old sociology, in that we desert verbal discussion for statistical facts; (2) We apply the new methods of statistics, which form a practically new calculus; (3) We start from three fundamental biological ideas: (*a*) That the relative weight of nature and nurture must not *a priori* be assumed, but must be scientifically measured; and thus far our experience is that nature dominates nurture, and that inheritance is more vital than environment. (*b*) That there exists no demonstrable inheritance of acquired characters. Environment modifies the bodily characters of the existing generation, but does not modify the germ plasm from which the next generation springs. (*c*) That all human qualities are inherited in a marked and probably equal degree."

These statements we may take to be the fundamental conceptions of Eugenic doctrine, and before criticising them, it may be as well to pass on to the conclusions at which Professor Pearson inevitably arrives. In the first place it is clear that for him selection of good parents—by which term "good" we must imply vaguely those with qualities beneficial to national progress—is the only means of saving a nation from deterioration. By artificial means the selective force of death is impaired, and opportunity is given for those to reproduce their kind who, in a less enlightened society, would have succumbed at an early age. With regard to the first claim, we rather regret to find that this abstinence from discussion is not universal among the disciples of the school of "Eugenists." Already we have had one or two publications, written by a professional journalist, which are likely to do far more harm than good. The present form of Eugenic publications seems to answer its purpose adequately, and to us it is a vital point that they should be written by the people who are specially engaged in this work, and not by the facile pen of the dilettante. The second claim is not entirely satisfactory, for we all know how statistics can be used or abused. Nevertheless, we feel that Professor Pearson will be honest and merciful, as behoves a trained mathematician. The three fundamental biological ideas seem to present a wealth of controversy, which, we fear, could not be avoided. It is useless to revive old questions about the real meaning of "acquired characters." Lamarck himself particularly says that the theory of acquired characters does not apply to man, and even if it did, we should require data extending from at least two thousand years ago to the present day in order to prove any one case.

The theoretical aspect of Eugenics, so far as concerns the selective force of death, the differential birth-rate, and the instability of society as a whole, seems to rest upon very firm ground. But we do not think that the biological ideas can be accepted without reservations. On the whole, therefore, we are heartily in accord with Professor Pearson's view of the importance of Eugenics to the State, and the need for education of the general public in the elements of race-culture. There can be no doubt that the careful compilation of pedigrees, both of normal and of abnormal people under similar conditions, will be invaluable to future statesmen as a basis for legislative action. In the case of disease, the work is already in progress, and we trust that no field will be left uninvestigated in which valuable social information may be brought to light. But the work must of necessity be slow and very accurate before the conclusions of the laboratory can be translated into practical measures.

